# PD-1 Expression Status on CD8+ Tumour Infiltrating Lymphocytes Associates With Survival in Cervical Cancer

**DOI:** 10.3389/fonc.2021.678758

**Published:** 2021-06-04

**Authors:** Peiwen Fan, Xi Li, Yaning Feng, Hongchao Cai, Danning Dong, Yanchun Peng, Xuan Yao, Yuping Guo, Miaomiao Ma, Tao Dong, Ruozheng Wang

**Affiliations:** ^1^ The Third Affiliated Teaching Hospital of Xinjiang Medical University, Affiliated Cancer Hospital, Urumuqi, China; ^2^ Key Laboratory of Cancer Immunotherapy and Radiotherapy, Chinese Academy of Medical Sciences, Urumuqi, China; ^3^ CAMS Oxford Institute (COI), University of Oxford, Oxford, United Kingdom; ^4^ MRC Human Immunology Unit (HIU), MRC Weatherall Institute of Molecular Medicine, John Radcliffe Hospital, University of Oxford, Oxford, United Kingdom; ^5^ Key Laboratory of Oncology of Xinjiang Uyghur Autonomous Region, Urumuqi, China; ^6^ State Key Laboratory of Pathogenesis, Prevention and Treatment of High Incidence Diseases in Central Asia, Urumuqi, China

**Keywords:** PD-1, survial analysis, cellular diagnosis, HPV – human papillomavirus, cervical cancer

## Abstract

Despite the expansion of PD-1 checkpoint blockade to multiple types of cancer, whether the programmed cell death 1 (PD-1) expression status on CD8+ tumour infiltrating lymphocytes (TILs) could be a prognostic factor in cervical cancer is still unclear. In this study, we performed *ex vivo* phenotypic analysis of PD-1 expression on CD8+ TILs by flow cytometry from 47 treatment-naïve cervical cancer patients. With a median follow-up of 26.1 months (95% confidence interval [CI], 24-28.2 months), we then linked the quantitative cellular expression results to progression-free survival and overall survival. Based on the intensity of PD-1 expression, we further categorised the cervical cancer patients into PD-1^high^ expressers (29.8%, 14/47) and PD-1^low^ expressers (70.2%, 33/47). Multivariate analysis revealed that PD-1^high^ expressers are correlated with early recurrence (HR, 5.91; 95% CI, 1.03-33.82; P= 0.046). Univariate analysis also demonstrated that PD-1^high^ expressers are associated with poor overall survival in cervical cancer (HR, 5.365; 95% CI, 1.55-18.6; P=0.008). Moreover, our study also demonstrated that CD8+/CD4+ TIL ratio and HPV infection status are risk factors for early relapse and mortality in cervical cancer patients. In conclusion, this study confirms that PD-1 expression status is an independent prognostic factor for progression free survival in cervical cancer. These findings could be important in predicting the relapse of cervical cancer as a cellular diagnosis method and could be important knowledge for the selection of prospective PD-1 blockade candidates.

## Introduction

Targeting the pathways of immune checkpoint receptors (ICRs) on tumour infiltrating lymphocytes (TILs) and their ligands in the tumour microenvironment revolutionized the way we treat advanced stage cancers (1). With the approval of ipilimumab by FDA, representing the first checkpoint inhibitor for metastatic melanoma, the era of checkpoint blockade in cancer treatment has officially started (2). However, the application of CTLA-4 blockade is very limited due to the high grade adverse effects and non-specific immune activation it causes (3, 4). It is thought that the benefits CTLA-4 inhibitor brings are proportional to the magnitude of immune tolerance it releases (5). PD-1/PD-L1 axis blockades demonstrate noteworthy benefits in treating multiple types of cancer including melanoma, head and neck cancer, bladder cancer, lung cancer and triple negative breast cancer with manageable adverse effects (6–10). Currently there are 3 approved PD-1 inhibitors, namely nivolumab, pembrolizumab and cemiplimab; and 3 approved PD-L1 inhibitors, atezolizumab durvalumab and avelumab for treating multiple types of cancer (11, 12).

The research of PD-1/PD-L1 blockades in cervical cancer initiated from 2015 onwards, of which 4 studies publicized their results with 2 phase I trials, 1 phase II trial and 1 phase I-II trial. The overall response rate (ORR) reported from those checkpoint blockade studies targeting PD-1/PD-L1 axis ranged from 17% to 27% (13–16). In 2018, FDA subsequently approved pembrolizumab for the treatment of advanced cervical cancer with disease progression during or after chemotherapy (11).

PD-1 and other ICRs expression on T-cells have contradictory roles in the immune regulation. On the one hand, it is shown that the increased PD-1 expression on T cells represents a more exhausted phenotype with impaired T-cell functionality against cancers or chronic virus infection (17, 18). On the other hand, the upregulation of PD-1 and other ICRs is also linked to antigen experience, T-cell activation and T-cell differentiation (19, 20). Moreover, PD-1 positive TILs has been suggested to be a favourable prognostic factor in HPV-associated head and neck cancer (21). Therefore, it is crucial to further explore the role of ICRs on T-cells in immune modulation and their potential application as biomarkers.

Little is known about the association between the expression of key ICRs on CD8+ TILs and prognosis in cervical cancer. We have previously described the dominant expression patterns among multiple ICRs on TILs and shortlisted PD-1 and Tim-3 as the key ICRs on TILs which may associate with clinical outcomes in multiple types of cancer (22). With the identification and characterization of PD-1^high^ and PD-1^low^ T cell subpopulations in the tumour from multiple cancer types including lung cancer, hepatocellular carcinoma and nasopharyngeal carcinoma, we believe that it is important to evaluate if the PD-1 expression status on CD8+ TILs associates with the recurrence or the overall survival of cervical cancer patients (23–25). Therefore, in this study, we hypothesize that (1) either the intensity of PD-1 expression (PD-1high VS PD-1low) or the frequency of key ICRs (PD-1 and Tim-3) on CD8+ TILs may have a prognostic value as a cellular biomarker in cervical cancer patients; (2) CD8+ and CD4+ TIL ratio is linked to the outcome of cervical cancer. Therefore, together with all the available clinical characteristics, we will conduct a systemic approach to determine what clinical factors or cellular markers on TILs can be used to predict the prognosis of cervical cancer patients.

## Material and Methods

### Cohort and Study Subjects

From 2017 to 2018, 47 cervical cancer patients were recruited in the cohort for the survival study in Xinjiang Tumour Hospital with a clinical diagnosis of stage I to stage III. Patients had undergone radiotherapy, combination of radiotherapy and chemotherapy or surgical treatment based on their clinical status. Written informed consent was given from all cancer patients. Prior to the recruitment in the cohort, all the cervical cancer patients were treatment-naïve and had no history of receiving any courses of HPV vaccines. Histopathological features of the tumour were examined in the Pathology Department in Xinjiang Tumour Hospital. All methods were performed in accordance with the relevant guidelines and regulations. The ethics committee of the Third Affiliated Hospital of Xinjiang Medical University Ethics Committee approved this study. Ethical approval was obtained from the Oxford Radcliffe Biobank (ORB) research tissue bank ethics committee (OCHRE reference 17/A006; REC reference 09/H0606/5+5), Oxford Tropical Research Ethics Committee (OxTREC Reference: 587-16).

### Isolation of Tumour Infiltrating Lymphocytes From Tumour Samples

Surgical or biopsy tumour tissues from cervical cancer patients were immediately transferred to tumour dissociation solution-containing (miltenyi biotec, catalog No. 130-095-929) C tube (miltenyi biotec, catalog No. 130-093-237). The tissues were then dissected into 1-3mm pieces by sterile surgical scissor (Ethicon, USA). C tubes were placed on Octo-gentle dissociator (miltenyi biotec, catalog No. 130-095-937). Human tumour program-1 was performed for the dissociation followed by 20mins incubation on the Gentle-mix rotator (miltenyi biotec, catalog No. 130-090-753) at 37°C, 5% Co2 incubator. 70nm cell strainer (Sigma-Aldirch, Dorset, UK) was then applied to purify the intra-tumoural lymphocytes. Further, cells were washed twice in R10 and counted by trypan blue staining.

### Multichromatic Flow Cytometry Staining

6-color panels were designed for *ex vivo* phenotypic analysis. After the dissociation of tumour samples, TILs were each initially stained with LIVE/DEAD^®^ Fixable Aqua Dead Cell Stain Kit (ThermoFischer Scientific) for 20 mins before surface staining with conjugated antibodies in Fluorescence-activated cell sorting (FACS) washing buffer (Phosphate-buffered saline (PBS) with 0.5M Ethylenediaminetetraacetic acid (EDTA) and 7.5% Bovine serum albumin (BSA) solution) for another 20 mins and fixed with 1x CellFix solution (BD Biosciences). Commercial conjugated antibodies used include CD3-Alexa Fluor 700 (344822, Biolegend), CD4-FITC (345768, BD Biosciences), CD8-APC-Cy7 (560179, BD Biosciences), PD-1-BV650 (564104, BD Biosciences), Tim3-BV421 (345008, Biolegend). The antibody cocktails were tested in advance with or without the use of tumour dissociation solution to ensure proper function. Fluorescence minus one (FMO) controls were applied accordingly in order to properly position gates. Flow cytometry in this study was conducted using a 4-laser BD LSR Fortessa flow cytometer. Quality check of the cytometer lasers and fluidic system was conducted on a daily basis prior to experiments. After excluding dead cells and doublets, we then selected CD3+ TILs and further gated CD4+ and CD8+ TILs, respectively. Fluorescent minus One (FMO) was applied to facilitate proper gating. Antibodies were titrated by PBMCs from healthy donors in Weatherall Institute of Molecular Medicine, University of Oxford prior to the experiment. In order to ensure the quality of FACS data, we ruled out any tumour samples in which the viable CD3+ TILs were lower than 10,000 cells. Full gating strategy of the 6-color FACS panel was attached in **Supplementary Table 1**. The exemplary gates of PD-1 and Tim-3 on T cells from tumour samples are shown in **Supplementary Figures 1A, B**.

### Defining High CD8+/CD4+ TIL Ratio Group and Low CD8+/CD4+ TILS Ratio Group

The ratio of CD8+ versus CD4+ TILs was analysed for all patients. Considering the sample size of this study, we ranked the CD8+/CD4+ ratio of all patients in the cohort and used the median CD8+/CD4+ ratio value as the cut-off to define the CD8+/CD4+ high ratio group and CD8+/CD4+ low ratio group. The range of CD8+/CD4+ TIL ratios in patients from this cohort is 0.72 ± 0.80.

### HPV Detection and Genotyping

Clinical kit appointed by the Cervical Cancer Prevention Program in China was used for HPV detection and genotyping in our study. Cervical swab samples were collected during vaginal or colposcopy examination. Hybribio Female Sample Collection Kit (HBCK-F) was used to collect liquid based cytology specimens. 21 HPV GenoArray Diagnostic Kit (HBGA-21PKG) was applied to detect HPV and genotyping. Hybribio diagnostic kit is a Polymerase Chain Reaction (PCR) based test, which amplifies extracted HPV DNA from cervical samples. Amplicons are then hybridized with specific HPV probes in the kit following by immunoassay method to achieve colourimetric results. HPV genotyping in the kit includes 15 high risk types: HPV 16, 18, 31, 33, 35, 39, 45, 51, 52, 53, 56, 58, 59, 66 and 68 and 6 low risk types: HPV 6, 11, 42, 43, 44 and 81. 10 patients in our cohort were negative according to this kit test (negative for all 21 genotypes) when recruited in this study. 37 patients tested positive for high risk genotypes in our cohort. All patients were treatment-naïve prior to HPV testing.

### Statistical Analysis

The PRISM 8 and SPSS 26 software were used for statistical analyses and graph plotting. Categorical variables were presented as absolute numbers and percentages, which were compared by Chi-square exact test for significance. Numeric variables were expressed as mean and standard deviation, which were compared by t test for significance. Overall survival was defined from the time of diagnosis in their first visit in Xinjiang Tumour Hospital to the recorded date of death. Progression free survival was defined from the date of receiving clinical treatments against cancer to the date of local or distant relapse confirmed by radiological examination. Survival rates were analysed by Kaplan-Meier method. The significance (0.05) of Kaplan-Meier curves was compared by log-rank test. Univariate and multivariate cox models were applied to analyse the prognostic factors (PD-1 status, CD8 VS CD4 TIL ratio, PD-1 expression level on CD8 TILs and Tim-3 expression level on CD8 TILs, tumour stage, tumour size, histological classification, HPV infection status and lymph node metastasis). Hazard ratios with a 95% confidence internal were calculated for the estimation of risk.

## Results

### Identification of PD-1^high^ and PD-1^low^ Expressers

**Figure 1:**
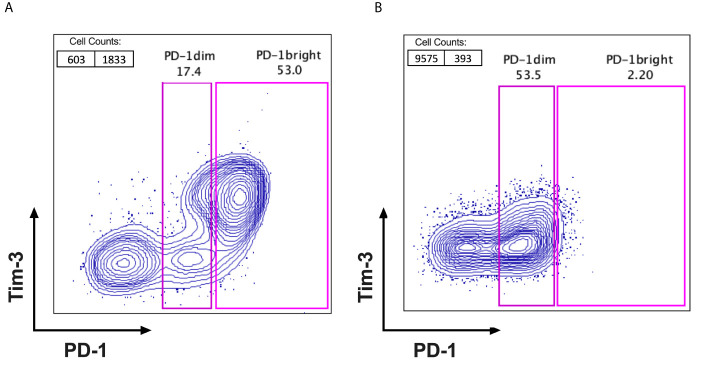
Identification of PD-1^high^ expressers and PD-1^low^ expressers. FACS plots with PD-1 (X axis) VS Tim-3 (Y axis) were gated on CD3+ and CD8+ TILs after excluding the dead cells from fresh surgical or biopsy tumour samples of cervical cancer patients. Based on the fluorescent intensity of PD-1 on CD8+TILs, patients can be cellularly sub grouped as **(A)** PD-1^high^ expressers; with 2 distinct PD-1 populations observed on FACS dot plots (PD-1^dim^ and PD-1^bright^) and the percentage of PD-1^bright^ subpopulation is higher than that of PD-1^dim^ subpopulation and **(B)** PD-1^low^ expressers; with only one PD-1 positive population visible on FACS dot plots and the percentage of PD-1^bright^ is lower than that of PD-1^dim^ subpopulation.

When analysing the frequencies and the expression pattern of PD-1 on CD8+ lymphocytes, subpopulations of PD-1+ CD8 TILs (PD-1^bright^ and PD-1^dim^) from tumour samples were observed in the FACS data from cervical cancer patients. We categorized all the cervical cancer patients in our cohort into 2 subgroups based on their distinct PD-1 expression status. PD-1^high^ expressers are defined as patients with higher frequency of PD-1^bright^ subpopulation on CD8+ TILs when compared to that of PD-1^dim^ subpopulation on CD8+ TILs (**Figure 1A**). PD-1^low^ expressers are defined as patients with lower frequency of PD-1^bright^ subpopulation than that of PD-1^dim^ subpopulation on CD8+ TILs (**Figure 1B**). The detailed PD-1 expression profiling on CD8 TILs in all cervical patients from our cohort is listed in **Supplementary Figure 3**, where the frequencies of PD-1- CD8 TILs, PD-1^dim^ CD8 TILs and PD-1^bright^ CD8 TILs are compared in each individual patient.

### PD-1^high^ Expression Status Positively Correlates With Lymph Node Metastasis

14 out of 47 cervical cancer patients are PD-1^high^ expressers, accounting for 29.8% of patients in our cohort. In contrast, 70.2% (33/47) of patients in the cohort are PD-1^low^ expressers. It is noticeable that among all the clinical characteristics listed in **Table 1**, it is more frequent for PD-1^high^ expressers (**P=0.004) to be diagnosed with lymph node metastasis (78.6%) compared to PD-1^low^ expressers (30.3%). The average age of cervical cancer patients in our study is 54.9, with no statistical difference (p=0.73) between PD-1^high^ expressers and PD-1^low^ expressers on the basis of age. The proportions of patients in our cohort with stage I, stage II and stage III cervical cancer are 14.9% (7/47), 40.4%(19/47) and 44.7%(21/47), respectively. The majority of patients (66%, 31/47) in our study were diagnosed with large tumours above 4cm in diameter, relative to 34% of patients with tumours less than 4cm in diameter. No statistical difference in cancer stage or tumour size was observed between PD-1^high^ expressers and PD-1^low^ expressers, although PD-1^high^ expressers have higher proportions of late tumour stage (64.3%) and large tumour size (85.7%) when compared to that of PD-1^low^ expressers (36.4% and 57.6%, respectively). In the category of histological stratification, no patients were diagnosed with well-differentiated cancer type in our cohort. However, more than half of the patients were diagnosed with moderately-differentiated cancer type (59.6%, 28/47) compared to 40.4% (19/47) of patients with poorly-differentiated cancer type. The majority patients in our study have elevated squamous cell carcinoma antigen (SCC) level (83%, 39/47), of which no statistical difference was observed between PD-1^high^ expresser and PD-1^low^ expresser groups.

**Table 1:** Clinical and histopathological characteristics of cervical patients in the cohort.

	Total patients(n=47)	PD-1 high expressers(n=14)	PD-1 low expressers(n=33)	p value
**Age**	** **			0.73
Mean	54.9	54.88	55	
Standard deviation	10.4	10.69	10.08	
**FIGO stage**	** **			0.2
Stage I	7 (14.9)	1 (7.1)	6 (18.2)	
Stage II	19 (40.4)	4 (28.6)	15 (45.4)	
Stage III	21 (44.7)	9 (64.3)	12 (36.4)	
**Tumor size**	** **			0.09
<4cm	16 (34)	2 (14.3)	14 (42.4)	
>4cm	31 (66)	12 (85.7)	19 (57.6)	
**Histological stage**	** **		s	0.19
poorly differentiated	19 (40.4)	8 (57.1)	11 (33.3)	
moderately differentiated	28 (59.6)	6 (42.9)	22 (66.7)	
**Histological classification**				0.35
Squamous cell carcinoma	45 (95.7)	14 (100)	31 (94)	
Adenocarcinoma	2 (4.3)	0 (0)	2 (6)	
**HPV status**				0.12
Positive	37 (78.7)	9 (64.3)	28 (84.8)	
Negative	10 (21.3)	5 (35.7)	5 (15.2)	
**SCC**	** **			0.21
Normal level	8 (17)	4 (28.6)	4 (12.1)	
Elevated level	39 (83)	10 (71.4)	29 (87.9)	
**Lymph node metastasis**	** **			**0.004
With metastasis	21 (44.7)	11 (78.6)	10 (30.3)	
Without metastasis	26 (55.3)	3 (21.4)	23 (69.7)	
**Treatment**	** **			0.43
Radiotherapy	17 (36.2)	5 (25.7)	12 (36.4)	
Radiotherapy+chemothrapy	18 (38.3)	7 (50)	11 (33.3)	
surgery	12 (25.5)	2 (14.3)	10 (30.3)	

The age, tumour stage, tumour size, cancer differentiation stage, histological classification, HPV status, lymph node metastasis and treatment options are compared between PD-1^high^ expressers and PD-1^low^ expressers. P value of numeric variable (age) was calculated by t test and P value of categorical variable was calculated by chi square test. In the comparison of age between 2 groups of patients (PD-1^high^ and PD-1^low^ expressers), mean and standard deviation were calculated.

### Identification of Independent Factors for Disease Relapse in Cervical Cancer

**Figure 2:**
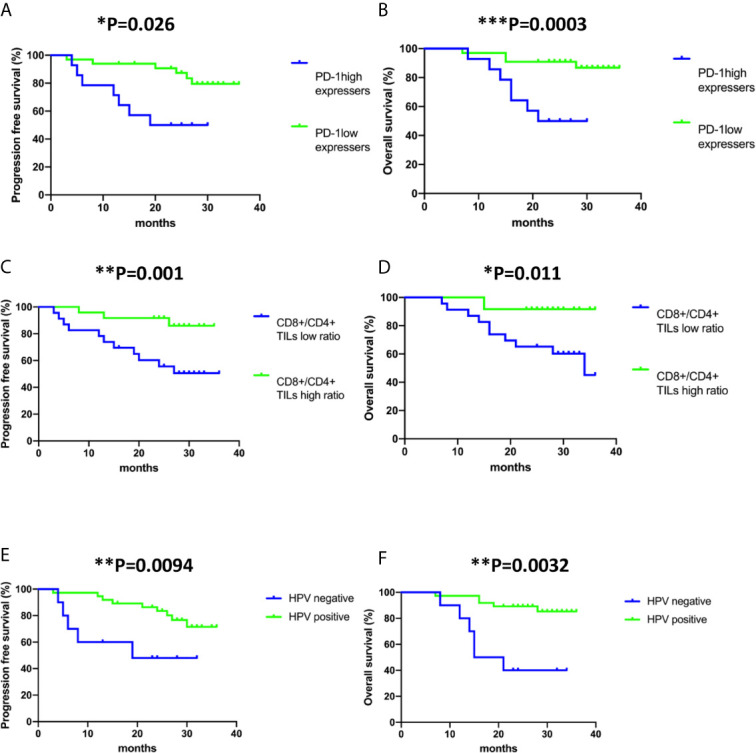
Kaplan–Meier analysis of PD-1 expression status on CD8+ TILs, CD8+/CD4+ ratio and human papillomavirus (HPV) status in all the cases included in the study. **(A, C, E)** Progression-free survival (time from diagnosis to the first local recurrence or metastasis); **(B, D, F)** Overall survival (time from the date of diagnosis to the date of death). Deaths without documented progression were censored at the date of death. Survival curves were compared using the log-rank test.

At a median follow-up of 26.1 months (95% confidence interval (CI) 24-28.2 months months),13 out of 47 patient (27.7%) had developed relapse and the death of 11 out of 47 patients (23.4%) had been reported. In univariate analyses, progression-free survival was significantly associated with multiple factors including tumour stage (hazard ratio (HR), 4.85; 95% CI, 1.47-16; P= 0.01), tumour size (HR, 8.63; 95% CI,1.47-16; P= 0.039) HPV negativity (HR, 3.82; 95% CI, 1.23-11.92; P=0.021), lymph node metastasis (HR, 3.44; 95% CI, 1.05-11.7; P= 0.041), PD-1 expressing status (HR, 3.89; 95% CI, 1.29-11.71; P=0.016) and CD8+/CD4+ TIL ratio (HR, 7.1, 95% CI, 1.57-32.09; P=0.011). In multivariate analysis, HPV negativity (HR, 12.367; 95% CI, 1.30-117.05; P= 0.028), PD-1^high^ expression status (HR, 5.91; 95% CI, 1.03-33.82; P= 0.046) and low CD8+/CD4+ TIL ratio (HR, 22.498; 95% CI, 3.66-138.39; P= 0.001) correlated with early recurrence in cervical cancer (**Table 2**). The 2-years progression-free survival was 50% for patients with PD-1^high^ expression status, but was 87.34% for patients with PD-1^low^ expression status (**Figure 2A**). The 2-years progression-free survival of patients with high CD8+/CD4+ TIL ratio was 91.3%, versus 55.65% in patients with low CD8+/CD4+ TIL ratio (**Figure 2C**). 2-years progression-free survival rates were 48% and 81.6% between HPV-negative patients and HPV-positive patients, respectively (**Figure 2E**).

**Table 2:** Univariate and multivariate cox proportional hazard models for relapse.

Variable	HR for relapse	95% CI	p value	Sig
**Univariate analysis**				
Stage	4.85	1.47-16	0.01	√
Lymph node metastasis	3.444	1.05-11.7	0.041	√
Tumour size	8.63	1.12-66.48	0.039	√
Negative HPV status	3.823	1.23-11.92	0.021	√
Histology	1.39	0.43-4.5	0.587	
Low CD8+/CD4+ TILs ratio	7.102	1.57-32.09	0.011	√
PD-1 expressing status on CD8 TILs	3.89	1.29-11.71	0.016	√
PD-1 positive on CD8+ TILs (%)	1	0.97-1.04	0.746	
Tim-3 positive on CD8+ TILs (%)	0.995	0.97-1.02	0.665	
**Multivariate analysis**				
Stage	4.831	0.85-27.41	0.075	
Lymph node metastasis	2.242	0.529.72	0.281	
Tumour size	13.906	0.43-453.54	0.139	
Negative HPV status	12.367	1.30-117.05	0.028	√
Histology	3.47	0.34-35.14	0.292	
Low CD8+/CD4+ TILs ratio	22.498	3.66-138.39	0.001	√
PD-1 expressing status on CD8 TILs	5.91	1.03-33.82	0.046	√
PD-1 positive on CD8+ TILs (%)	1.011	0.97-1.06	0.64	
Tim-3 positive on CD8+ TILs (%)	0.993	0.97-1.02	0.546	

Univariate and multivariate Cox Proportional Hazard Models were applied to identify the potential factors that may predict the relapse of disease after treatment. Values of HR (Hazard ratio) and 95% CI (confidence interval) were listed. Factors with P value < 0.05 were marked a tick in the significance (Sig) column. Orange colour variables are clinical parameters and blue colour variables are cellular factors.

### HPV-Negativity, Late Stage Cancer Type and Low CD8+/CD4+ TIL Ratio Are Correlated With Poor Overall Survival

In the investigation of risk factors for mortality in cervical cancer, both univariate and multivariate analyses revealed that late stage of diagnosis (HR, 6.062; 95% CI, 1.43-25.69; P= 0.014 and HR, 12.397; 95% CI, 1.43-25.69; P= 0.043, respectively), HPV negativity (HR, 6.744; 95% CI, 2.02-22.47; P= 0.002 and HR, 15.663; 95% CI, 2.02-22.46; P= 0.011, respectively) and low CD8+/CD4+ ratio (HR, 5.272; 95% CI, 1.14-24.43; P= 0.034 and HR, 11.898; 95% CI, 1.14-24.43; P= 0.011, respectively) associate with shorter overall survival (**Table 3**). The 2-years overall survival rates of the high CD8+/CD4+ TIL ratio group and low CD8+/CD4+ TIL ratio group were 91.67% and 65.22%, respectively (**Figure 2D**). The 2-year overall survival of HPV-negative patients was 40% compared to 89.19% in HPV-positive patients (**Figure 2F**).

**Table 3:** Univariate and multivariate cox proportional hazard models for mortality.

Variable	HR for death	95% CI	p value	Sig
**Univariate analysis**				
Stage	6.062	1.43-25.69	0.014	√
Lymph node metastasis	3.708	0.98-13	0.053	
Tumour size	7.048	0.9-55.23	0.063	
Negative HPV status	6.744	2.02-22.47	0.002	√
Histology	0.715	0.22-2.35	0.581	
Low CD8+/CD4+ TILs ratio	5.272	1.14-24.43	0.034	√
PD-1 expressing status on CD8 TILs	5.365	1.55-18.6	0.008	√
PD-1 positive on CD8+ TILs (%)	0.984	0.95-1.02	0.31	
Tim-3 positive on CD8+ TILs (%)	0.994	0.96-1.03	0.709	
**Multivariate analysis**				
Stage	12.397	1.43-25.69	0.043	√
Lymph node metastasis	1.46	0.98-13	0.66	
Tumour size	4.296	0.9-55.23	0.349	
Negative HPV status	15.663	2.02-22.46	0.011	√
Histology	1.082	0.22-2.35	0.944	
Low CD8+/CD4+ TILs ratio	11.898	1.14-24.43	0.011	√
PD-1 expressing status on CD8 TILs	3.733	1.55-18.6	0.137	
PD-1 positive on CD8+ TILs (%)	0.992	0.95-1.02	0.614	
Tim-3 positive on CD8+ TILs (%)	0.993	0.96-1.03	0.746	

Univariate and multivariate Cox Proportional Hazard Models were applied to identify the potential factors that may predict the mortality of disease after treatment. Values of HR (Hazard ratio) and 95% CI (confidence interval) were listed. Factors with P value < 0.05 were marked a tick in the significance (Sig) column. Orange colour variables are clinical parameters and blue colour variables are cellular factors.

### PD-1^high^ Expressers Have Significantly Higher Risk for Disease Relapse

The independent effect of PD-1 expression status (PD-1^high^ VS PD-1^low^) of CD8+ TILs on progression-free survival and overall survival was evaluated by Cox proportional hazards regression models adjusted for tumour stage, tumour size, lymph node metastasis, HPV infection status, tumour pathohistological classification, CD8+/CD4+ TIL ratio, PD-1 frequency on CD8+ TILs and Tim-3 frequency on CD8+ TILs (**Supplementary Figure 2** and **Tables 2**, **3**). Both univariate and multivariate analyses confirmed that PD-1^high^ expressers are significantly associated with worse progression-free survival (HR, 3.89; 95% CI, 1.29-11.71; P= 0.016 and HR, 5.91; 95% CI, 1.03-33.82; P= 0.046, respectively). However, only univariate analysis but not multivariate analysis indicated that PD-1^high^ expressers are significantly correlated with poor overall survival (HR, 5.365; 95% CI, 1.55-18.6; P=0.008). Of note, the frequency of PD-1 or Tim-3 on CD8+ TILs had no association with either recurrence or mortality in cervical cancer (**Tables 2** and **3**). The 2-years overall survival was only 50% for patients with PD-1high expression status but was 90.9% for patients with PD-1^low^ expression status (**Figure 2B**). Therefore, PD-1 expression status has a prognostic value to predict relapse in cervical cancer patients.

## Discussion

Several HPV-negative cervical cancer studies demonstrated that HPV-negativity in cervical cancer correlates with poor disease-free survival and overall survival when compared to that of HPV-positive cervical cancer (26–28). However, these studies are based on Caucasian populations; little information between HPV infection status and prognosis of cervical cancer in Chinese population is known. Our study confirmed that HPV-negative cervical cancer patients have a higher risk for recurrence and shorter overall survival when compared to HPV-positive cervical cancer patients in China. Of note, in our cohort we did not observe increased frequency of adenocarcinoma in HPV-negative cervical cancer patients when compared to HPV-positive cervical cancer patients, which has been shown in previous research (29). In fact, the histological classification of all the HPV negative cervical cancer patients is squamous cell carcinoma and only 2 HPV positive patients are classified as adenocarcinoma in our cohort (**Table 1**).

Through *ex vivo* flow cytometric data obtained prior to clinical interventions of all the patients in the cohort, we have also investigated if the CD8+/CD4+ TIL ratio is a risk factor for cervical cancer. Our results indicated that increasing CD8+/CD4+ TIL ratio is a favourable prognostic factor, correlating with improved overall survival and delayed recurrence in cervical cancer. This result is in line with multiple previous studies in breast cancer, nasopharyngeal cancer, ovarian cancer and cervical cancer (30–33). It implicates that CD8+ TILs have a prominent role in controlling the progression of disease, with positive impact on overall survival, whereas the high percentage CD4+TILs in the tumour microenvironment may contribute to poor prognosis. Therefore, CD8+/CD4+ TIL ratio through biopsy can be a useful marker in predicting the outcome of cervical cancer and tuning the ratio of CD8+/CD4+ TILs by clinical intervention such as chemotherapy or radiotherapy may improve survival for patients. It is reported that in a colorectal cancer study that Naïve Tregs (CD3+CD4+FOXP3^low^CD45RA^+^) and effector Tregs (CD3+CD4+OXP3^high^CD45RA^−^) have immunosuppressive activity whereas non-Tregs (CD3+CD4+FOXP3^low^CD45RA^−^) have antitumour activity (34). However, our study only compared the bulk CD8+/CD4+ TILs in cervical cancer and is unable to distinguish Fr-III from bulk CD4+ TILs.

In the application of immune checkpoint receptor blockades, initially, it was thought that the increased frequency of ICRs such as PD-1 on TILs may be useful markers to grade the magnitude of T-exhaustion (35). However, the upregulation of PD-1 expression level is not just linked to T-cell exhaustion but is also associated with T-cell activation and T-cell differentiation in the cancer microenvironment and in the presence of chronic virus infection (36–39). This may partially explain why there is no association between the frequency of Tim-3 or PD-1 on CD8+ TILs and the recurrence or mortality of cervical cancer. Furthermore, we also investigated whether PD-1 expression has the potential to serve as a prognostic or a predictive biomarker for the recurrence and mortality of cervical cancer patients since no previous research on PD-1 expression status on T cells and its correlation with survival was reported in cervical cancer studies. Most of the PD-1 expression-related cancer research was conducted on paraffin-embedded samples, which may obscure its potential correlation with disease relapse or overall survival of cancer patients due to the difficulties of PD-1 expression quantification on paraffin blocks (40, 41).

In a liver cancer study, PD-1^high^ CD8+ TILs have been reported to exhibit a differential gene expression profile with a more exhausted T-cell functionality when compared to PD-1^low^ CD8+ TILs. However, PD-1^high^ CD8+ TILs are more functionally restored with a favourable response to PD-1 blockade compared to PD-1^low^ CD8+ TILs (23). In an HPV-associated nasopharyngeal cancer study, it is suggested that PD-1^high^ CD8+ TILs are associated with worse-disease free survival, and that HPV infection status may be a factor to determine the percentage of PD-1^high^ CD8+ TILs in NPC; a higher frequency of PD-1^high^ CD8+TILs with impaired anti-tumour functionality in HPV- tumours was detected when compared to that of HPV+ tumours (24). Based on PD-1 expression status on peripheral CD8 T cells, a recent/newly-published study in cervical cancer sub-divided patients into PD-1^high^, PD-1^int^ and PD-1^low^ subgroups and found significantly increased frequencies of PD-1^high^ and PD-1^int^ but not PD-1^low^ on CD8 T cells from cervical cancer patients when compared to healthy donors (42). These previous results in combination with our findings that PD-1^high^ expressers associate with early relapse in cervical cancer may indicate PD-1 expression status on CD8+ TILs not only has a prognostic value in disease recurrence but also may serve as a criterion to select patients for PD-1 blockade and cellular diagnosis.

To our knowledge, this is the first study analysing the correlation between PD-1 expression status on CD8 TILs and survival rates in cervical cancer patients. Indeed, our study demonstrated that PD-1 expression status, but not the frequency of PD-1 on CD8+ TILs, is an independent prognostic factor for progression free survival. PD-1 expression status may potentially be an independent prognostic factor for overall survival in cervical cancer as well since we observed that PD-1^high^ expressers have significantly higher mortality risk *via* the univariate analysis but not multivariate analysis.

## Data Availability Statement

The original contributions presented in the study are included in the article/**Supplementary Material**. Further inquiries can be directed to the corresponding authors.

## Ethics Statement

The studies involving human participants were reviewed and approved by ethics committee of the Third Affiliated Hospital of Xinjiang Medical University. The patients/participants provided their written informed consent to participate in this study.

## Author Contributions

TD, XL, and RZW: study design. PWF, YNF, XL, MMM, YPG, DND, HCC, YCP, and XY: data acquisition. XL, PWF, DND, and YPG: data analysis. XL, TD, and RZW: paper writing and edition. All authors contributed to the article and approved the submitted version.

## Funding

This work was supported by Nature Science Foundation of China(U1603282), the Key Laboratory of Cancer Immunotherapy and Radiotherapy, CAMS, China (grant no. 2019PT310021), Chinese Academy of Medical Sciences (CAMS) Innovation Fund for Medical Science (CIFMS), China (grant number: 2018-I2M-2-002).

## Conflict of Interest

The authors declare that the research was conducted in the absence of any commercial or financial relationships that could be construed as a potential conflict of interest.
