# Supplementary material for: PD-1 Expression Status on CD8+ Tumour Infiltrating Lymphocytes Associates With Survival in Cervical Cancer
Source: Front Oncol. 2021 Jun 4;11:678758. doi: 10.3389/fonc.2021.678758 (PMC8212040; doi:10.3389/fonc.2021.678758)
Supplement: Supplementary file 1 [file DataSheet_1.docx]

**Supplementary Figure 1: Gating strategy of PD-1 and Tim-3 on CD8 TILs**

**A:**

**Aqua live/dead**


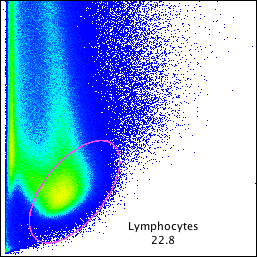

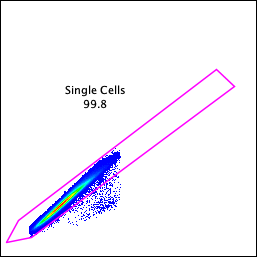

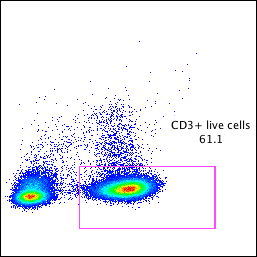

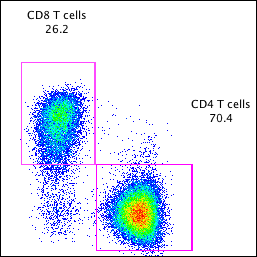


**CD8**

**FSC-H**

**SSC-A**

**FSC-A FSC-A CD3 CD4**

**B:**

**Gated on CD8+ TILs**

**
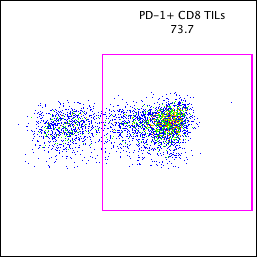

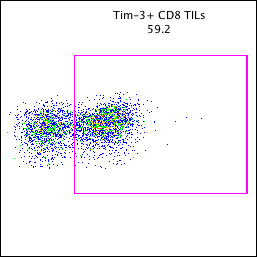
**

**CD8**

**CD8**

**PD-1 Tim-3**

**Supplementary Figure 1: A:** progressive gating strategy was used to exclude doublets and dead cells and to identify CD4 and CD8 T cells afterwards. **B:** The frequency of PD-1 and Tim-3 on CD8 TILs were gated as shown

**Supplementary Figure 2: Frequencies of PD-1 and Tim-3 on CD8 TILs**

**
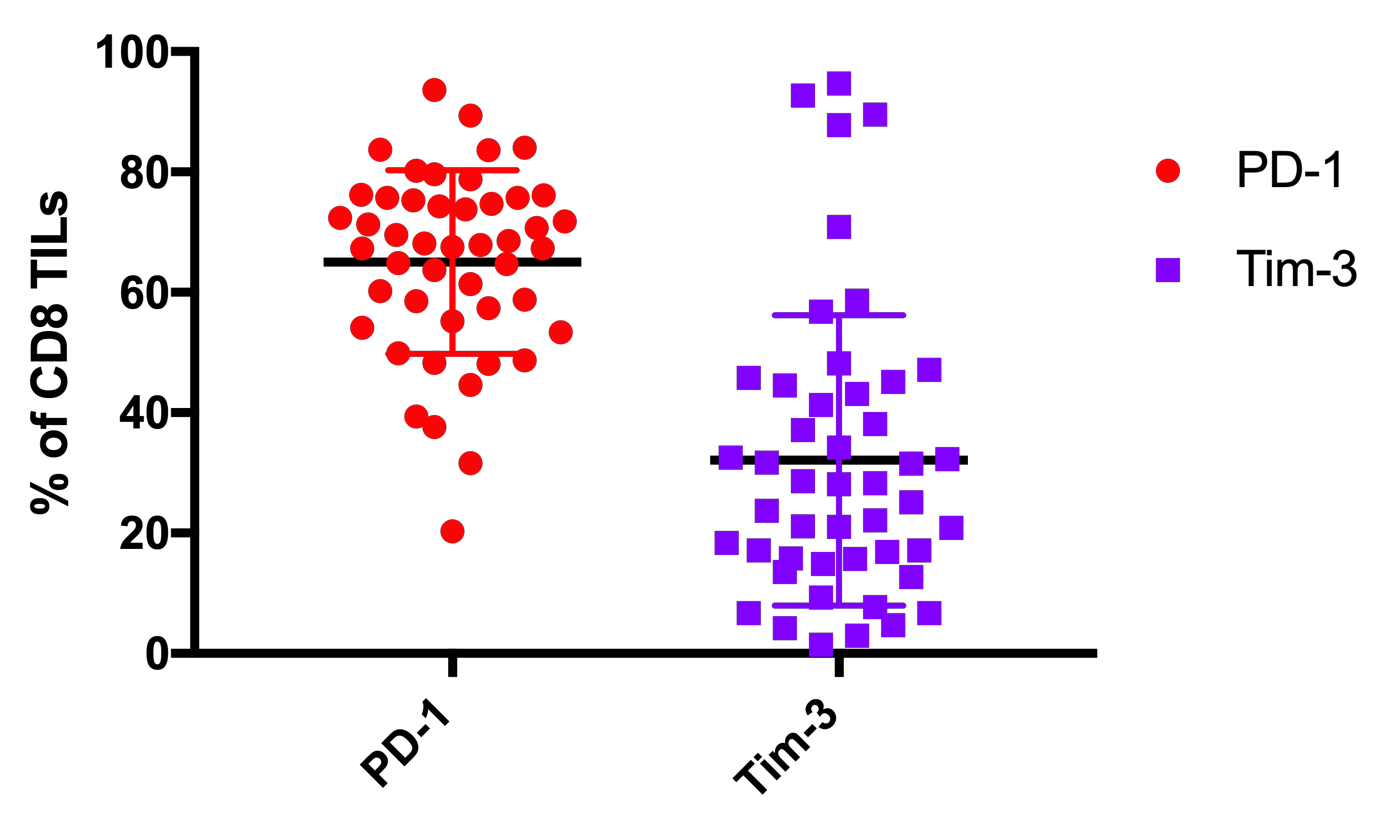
**

**Supplementary Figure 2:** After excluding doublets and dead cells and to identify CD4 and CD8 T cells afterwards, the frequencies of PD-1+ and Tim-3+ on CD8 TILs from 47 cervical cancer patients in our cohort were plotted as shown.

**Supplementary Figure 3: PD-1 expression profiling on CD8 TILs**

**
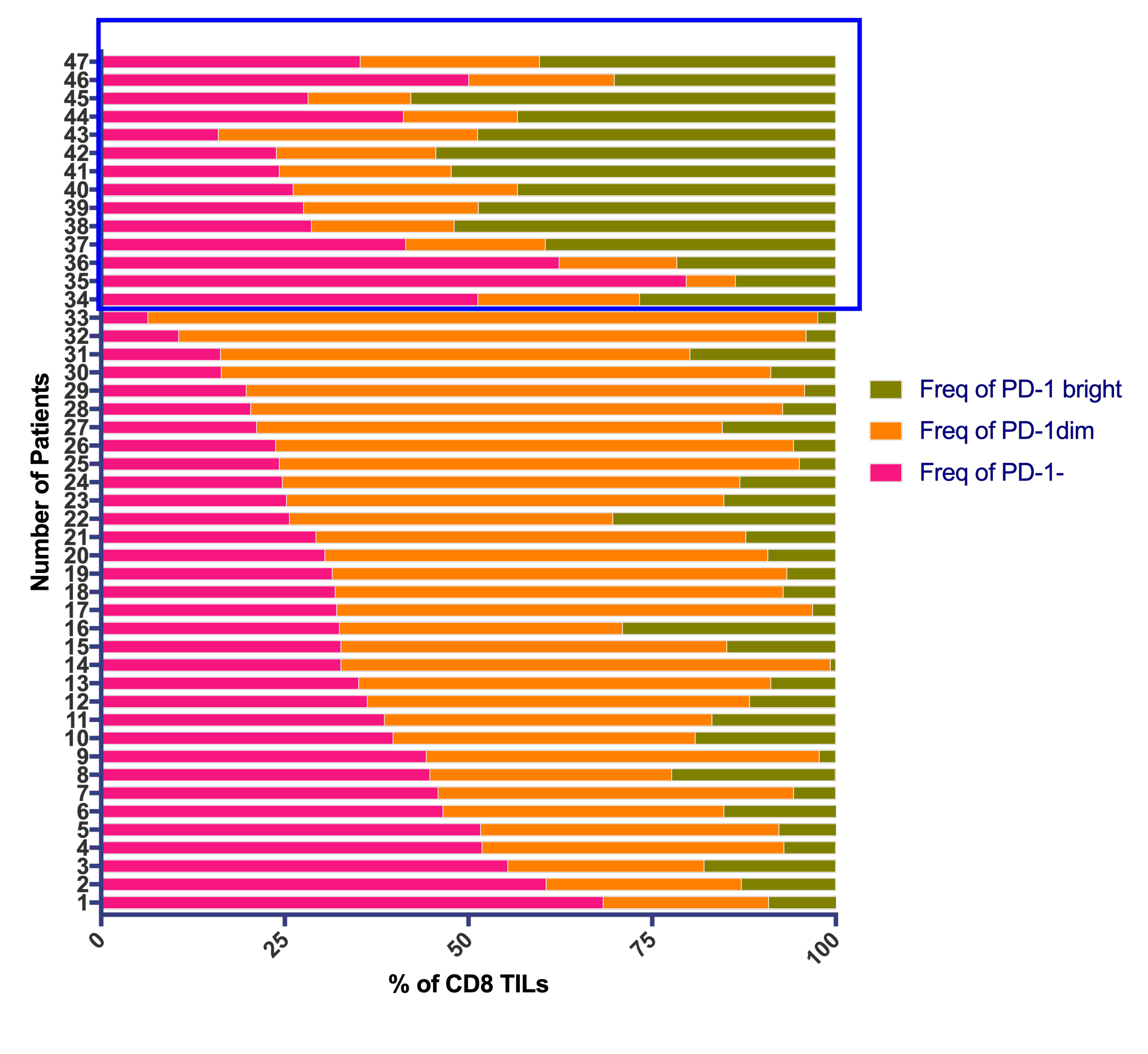
**

**Supplementary Figure 2:** PD-1- CD8 TIL (%, pink), PD-1 dim CD8 TIL (%, orange) and PD-1 bright CD8 TIL (%, green) are depicted for each patient; the blue rectangular box group (patient 34-47) is PD-1 high expressers and the rest bars are PD-1 low expressers (paient 1-33).
