# Supplementary material for: PD-1 Expression Status on CD8+ Tumour Infiltrating Lymphocytes Associates With Survival in Cervical Cancer
Source: Front Oncol. 2021 Jun 4;11:678758. doi: 10.3389/fonc.2021.678758 (PMC8212040; doi:10.3389/fonc.2021.678758)
Supplement: Supplementary file 2 [file DataSheet_2.docx]

**Supplementary Table 1:**

| **Fluorophore** | **Markers** |
| --- | --- |
| **BV421** | Tim-3 |
| **BV650** | PD-1 |
| **AF 700** | CD3 |
| **FITC** | CD4 |
| **APC-H7** | CD8 |
| **BV510** | L/D Aqua |

**Supplemntary Table 1:** The fluorophores of conjugated antibodies are listed in the left column and the cellular surface markers antibodies targeted are listed in the right column. The catalogue numbers of these commercial antibodies are detailed in material and method session.
